# Patient enrollment and logistical problems top the list of difficulties in clinical research: a cross-sectional survey

**DOI:** 10.1186/s12874-016-0151-1

**Published:** 2016-05-04

**Authors:** Stéphane Cullati, Delphine S. Courvoisier, Angèle Gayet-Ageron, Guy Haller, Olivier Irion, Thomas Agoritsas, Sandrine Rudaz, Thomas V. Perneger

**Affiliations:** Division of Clinical Epidemiology, Geneva University Hospitals, University of Geneva, Rue Gabrielle Perret-Gentil 6, CH–1211, Geneva 14, Switzerland; Division of Anesthesia, Department of Anesthesiology, Pharmacology and Intensive Care, Geneva University Hospitals, Geneva, Switzerland; Department of Gynecology and Obstetrics, Geneva University Hospitals, Geneva, Switzerland; Department of Clinical Epidemiology and Biostatistics, McMaster University, Hamilton, Canada

**Keywords:** Clinical research, Difficulties, Barriers, Medical researchers, Research protocols, Switzerland

## Abstract

**Background:**

Many medical research projects encounter difficulties. The objective of this study was to assess the self-reported frequency of difficulties encountered by medical researchers while conducting research and to identify factors associated with their occurrence.

**Methods:**

The authors conducted a cross-sectional survey in 2010 among principal investigators of 996 study protocols approved by the Research Ethics Committee in Geneva, Switzerland, between 2001 and 2005. The authors asked principal investigators to rate the level of difficulty (1: none, to 5: very great) encountered across the research process.

**Results:**

588 questionnaires were sent back (participation rate 59.0 %). 391 (66.5 %) studies were completed at the time of the survey. Investigators reported that the most frequent difficulties were related to patient enrollment (44.3 %), data collection (26.7 %), data analysis and interpretation (21.5 %), collaboration with caregivers (21.0 %), study design (20.4 %), publication in peer-reviewed journal (20.2 %), hiring of competent study personnel (20.2 %), and getting funding (19.2 %). On average, investigators reported 2.8 difficulties per project (SD 2.8, range 0 to 12). In multivariable analysis, the number of difficulties was higher for studies initiated by public sponsors (vs. private), single center studies (vs. multicenter), and studies about treatment, diagnosis or prognosis (i.e., clinical vs. other studies).

**Conclusions:**

Medical researchers reported substantial logistical difficulties in conducting clinical research.

**Electronic supplementary material:**

The online version of this article (doi:10.1186/s12874-016-0151-1) contains supplementary material, which is available to authorized users.

## Background

Evidence-based medicine [1] and clinical research are positively valued by health care professionals [2–5], many of whom engage in research [6–9] alongside their clinical activity, in many countries [7, 8] and in Switzerland [6]. Research is necessary to improve medical knowledge, and medical (or clinical) researchers pursuing an academic career have the incentive to publish their results so as to obtain credit from their peers and institutions. However, time devoted to research is generally scarce [9, 10] because clinical activity has priority. Furthermore, medical researchers report moderate levels of research skills [9, 11, 12]. As a result, the numbers of medical researchers [13, 14] and the number of applications for scientific career awards and for research funding have been decreasing over the past three decades, notably in the United States, Finland and Sweden [15–17]. This gap between the necessity of medical research and the limited ability of doctors to actually conduct medical research suggests the need to better understand the difficulties encountered by medical researchers.

Several difficulties in conducting clinical research have been already reported. Challenges include designing the study, calculating the adequate sample size, obtaining funding [18, 19], getting approval from the Research Ethics Committee (REC) [20–22], enrolling enough patients [23], performing appropriate statistical analyses [11] and writing up and publishing the findings [19, 24, 25]. Among discontinued clinical studies, four out of ten were not started because of lack of funding [26] and three out of ten were abandoned because of insufficient patient enrollment [26]. For randomized clinical trials, the ability to recruit patients during the first months after their initiation is a strong indicator for study completion and publication [25, 27]. Finally, getting the paper published in a peer-reviewed journal is also difficult: a quarter of submitted paper send up never being published [28].

While a number of aspects regarding study processes and outcomes have been investigated [25, 27–34], such as patient recruitment, completion rates, publication or number of citations, less is known about possible difficulties experienced during the course of a clinical study. Previous studies have explored the general impression of medical researchers about difficulties in research [19, 26, 35], but not the recall of actual difficulties experienced in the course of a specific study. Surveying medical researchers on a specific and completed study can provide a more accurate recall of the aspects of the research process that have actually been troublesome. We conducted therefore a survey among medical researchers of study protocols approved by a Swiss REC to assess the frequencies and types of difficulties experienced, and to examine factors associated with the occurrence of difficulties.

## Methods

### Setting

We defined medical researchers as principal investigators (PIs) of study protocols approved by the REC of the Geneva University Hospitals, Switzerland between January 1, 2001 and December 31, 2005. The Geneva University Hospitals is a single 1800-bed public teaching hospital, covering the entire range of medical disciplines, with eight sites across the Geneva region. About 1200 research protocols are currently signaled as ongoing by the REC.

### Study design and questionnaire

**Fig. 1:**
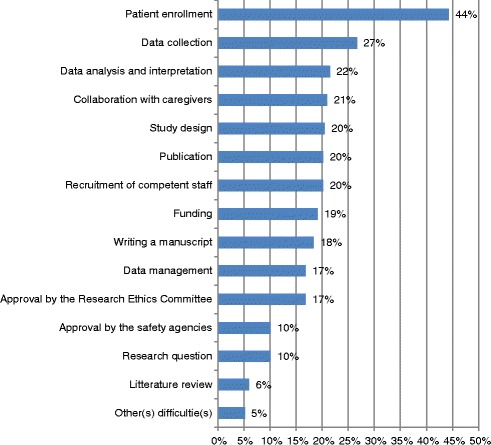
Self-reported difficulties* (rated as medium or great or very great) in clinical research among principal investigators (*N* = 386)

We conducted a cross-sectional survey among PIs of the selected protocols. We sent them a self-administered online questionnaire between November and December 2010, followed by two reminders. We asked PIs to answer one questionnaire for each separate protocol approved by the REC, identified by its title. The questionnaire had been pre-tested previously among of 12 medical researchers who were not part of the study. We asked PIs to report if the study was completed (defined as recruitment, data collection and analysis completed, whatever the study is published or not) or not (ongoing or abandoned) and whether they had received training in quantitative methods (none, continuing education at the University level, or bachelor/master/PhD in quantitative methods) at the time of the submission to the REC. If the study was completed, we asked PIs to state if they encountered any of 14 specific difficulties in the course of research (Fig. 1) on the following scale of difficulty: none, minor, medium, great, very great, or not applicable. Based on a review of the literature, a group of experts including methodologists (AGA, GH, SC, TA, TVP) and biostatisticians (DSC and Dr. Christophe Combescure, see acknowledgements) identified these 14 difficulties in conducting clinical research. The results from the pre-test with medical researchers suggested the difficulties were clearly described. “Other difficulties” could be reported in a fifteenth item as free text. We did not invite PIs to rate their difficulties with ongoing studies, as we wanted to include only hindsight evaluations of experience. We also asked PIs whether the results of the study were published or “in press”, to provide the reference in an open field, and also whether they had papers in submission or in preparation; for the latter studies, we updated the database by identifying relevant papers published up to November 2013 (PubMed search).

We identified characteristics of study protocols using information recorded in the archives of the REC. We abstracted data on study design (interventional, observational), first decision of the REC (positive, positive with recommendations, positive with written modifications required, positive with conditions and reassessment, negative, and not considered), public vs. private sponsorship of the study (i.e. hospital or university vs. industry or private foundation), link with the industry (none, industry with indication of support, industry without indication of support), planned sample size (later dichotomized as up to 100 vs. larger), reporting of sample size calculation (yes, no), number of centers (single versus multi center), clinical specialty (oncology, neurology, infectious disease, rheumatology, psychiatry, cardiology, general, other specialties, combination of two specialties), and presence of a statistician on study team (yes, no). The category “Other specialties” included all other specialties distributed across the medical services of the Geneva University Hospitals (65 medical services), or studies with no clear clinical specialty. We classified the type of research conducted into the following seven groups: study of health or illness (i.e., organs, tissues, cells, receptors, etc.), interventional or treatment, diagnosis, prognosis, public health or health economics or opinions or perceptions, study methods, and other. We also dichotomized this variable in study protocols informing clinical questions (i.e., interventional/treatment, diagnostic and prognostic studies) versus other types of research (i.e., studies of health/illness, public health, study methods, and other).

### Dependent variables

In the survey among PIs, each of the 14 items of self-reported difficulties was dichotomized into medium, great or very great difficulty (1) versus no or minor difficulty (0). When PIs answered “not applicable”, the value “0” (no difficulty) was assigned, because we considered not applicable difficulties as an absence of difficulty. The fifteenth item for “other difficulty(ies)” was followed by an open field for description. Some free-text responses from this item were similar to the other 14 difficulties already defined. One co-author (SC) recoded the text responses into the pre-defined difficulties and another co-author (TP) checked the recoding. Eight free-text responses were re-classified and 41 remained in the “other” category.

Dichotomized self-reported difficulties were used to calculate sums of difficulties for each study and then to compare mean numbers of difficulties across study characteristics.

We defined three dependent variables. The first was a score summing all difficulties across the whole research process. We then classified the 14 difficulties into dimensions using factor analysis with varimax rotation (for a complete description, see Additional file 1: Appendix 1). The analysis suggested two dimensions of seven difficulties each: a dimension related to “scientific difficulties” (literature review, research question, study design, data management, data analysis, manuscript writing, publication in a peer-reviewed journal) and a dimension related to “logistical difficulties” (approval by the REC, getting funding, getting approval by safety agencies, patient enrollment, data collection, collaboration with caregivers for patient enrollment, recruiting competent staff). Dimension scores based on the sum of corresponding items were the second and third dependent variables. Both had adequate internal consistency, with a Cronbach alpha coefficient of 0.83 for scientific difficulties and 0.73 for logistical difficulties.

### Sample size calculation

The analysis of difficulties encountered by researchers was a secondary objective of this study. The primary objective of this study was concerned with publication of clinical studies (manuscript in preparation) and sample size was determined with regard to the primary objective. However, a sample size of 400 protocols yielded the following precision (exact 95 % confidence interval) in the estimation of prevalence for a given difficulty: 5.0 % (3.1 to 7.6 %), 10.0% (7.2 to 13.4 %), 20 % (16.2 to 24.3 %), 30% (25.5 to 34.8 %), and 40 % (35.2 to 45.0 %). A comparison of means of 2 groups of 200 observations each would allow for the detection of a small effect size (0.28) with an alpha of 0.05 and a power of 80 %.

### Statistical analysis

The unit of analysis was the study protocol. We treated the three dependent variables (sum of all difficulties, sum of scientific difficulties and sum of logistical difficulties) as continuous. We first compared the means across the study characteristics using one-way ANOVA. Then a linear regression model was constructed to predict the mean scores of difficulties using predictors that were statistically significant in univariable analysis (at *p* ≤ 0.05). We conducted all analyses with SPSS version 22.0 (IBM Corporation, Armonk, United States).

## Results

### Sample

Among the 996 study protocols, 588 questionnaires were returned by the PIs (participation rate 59.0 %). Among these 588 studies, 391 (66.5 %) were completed at the time of the survey, 96 (16.3 %) were still ongoing and 101 (17.2 %) had been abandoned.

More than three of four completed protocols were sponsored by a public institution, about two thirds were published, a majority was single center studies, and about half were observational studies (Table 1). The median of the sample sizes was 100 (mean 591, interquartile range 260, range 1 to 25,000) and a majority of study protocols did not report sample size calculation. Study protocols reporting a sample size calculation had more often a sample size above the median compared to study protocols that did not (60.9 % versus 30.4 %, *p*-value <0.001). Almost three protocols out of four did not have a statistician in the study team. Most PIs reported training in quantitative methods – courses or continuing education or a University degree –at the time of the protocol submission to the REC.

**Table 1:** Characteristics of the 391 completed studies with follow-up questionnaire returned

	*N* (%)
Study design	
Interventional	180 (46.0)
Observational	211 (54.0)
First decision of the research ethics committee (*N* = 390)	
Positive, positive with recommendations, or positive with written modifications required	309 (79.2)
Positive with conditions and reassessment, or negative and non-considered, but approved at a later stage	81 (20.8)
Financial sponsorship of the study protocol (*N* = 377)	
Public (hospital, university)	294 (78.0)
Private (industry, foundation)	75 (19.9)
Both	8 (2.1)
Link with the industry (*N* = 378)	
None	267 (70.6)
Industry with indication of support (funding, drugs, human resources)	103 (27.2)
Industry without indication of support	8 (2.1)
Sample size (*N* = 390)	
≤ 100	220 (56.4)
> 100	170 (43.6)
Sample size calculation (N = 384)	
Yes	169 (44.0)
No	215 (56.0)
Number of centers	
Single	232 (59.3)
Multicenter	159 (40.7)
Research topic	
Health or illness (organs, tissues, cells, receptors, etc.)	91 (23.3)
Intervention or treatment	172 (44.0)
Diagnostics method	25 (6.4)
Prognosis	12 (3.1)
Public health, health economics, medical ethic, attitudes	43 (11.0)
Research methods (questionnaires, indicators, measures, etc.)	41 (10.5)
Other, unspecified topic (registries, medical record databases)	7 (1.8)
Clinical specialty	
General	60 (15.3)
Psychiatry	40 (10.2)
Cardiology	35 (9.0)
Oncology	30 (7.7)
Infectious diseases	27 (6.9)
Neurology	16 (4.1)
Rheumatology	7 (1.8)
Other clinical specialties	149 (38.1)
Two or more specialties	27 (6.9)
Presence of a statistician in the study team (*N* = 384)	
Yes	98 (25.5)
No	286 (74.5)
PI’s training in quantitative methods	
None	164 (41.9)
Courses and/or continuing education at the University level	183 (46.8)
Bachelor, master or PhD in quantitative methods	8 (2.0)
Bachelor, master, or PhD in quantitative methods, and courses/continuing education	36 (9.2)
Study published	
Yes	269 (68.8)
No	122 (31.2)

For 408 study protocols the questionnaire was not returned by the PI. Information on these 408 studies was collected using their study protocol stored in the archives of the REC. More than seven of ten studies had no statistician in the study team, around two third had public funding, and the majority had no link with the industry, were single center studies, were unpublished and had an interventional design (Additional file 1: Appendix 2). The median sample size was 100 (mean 973, interquartile range 321, range 3 to 136,000) and a majority of study protocols did not justify sample size. Compared with included studies, studies with no questionnaire had higher proportions of interventional studies, private sponsorship, link with the industry and were more frequently unpublished.

### Difficulties in clinical research and associated study characteristics

Levels of difficulties are reported in Fig. 1: patient enrollment and data collection were the most frequently reported. The mean number of difficulties was 2.8 (standard deviation (SD) 2.8, min 0, max 12); 26.4 % of PIs reported no difficulty and 18.7 % reported one difficulty only (Additional file 1: Appendix 3). The mean number of scientific difficulties was 1.1 (SD 1.7, min 0, max 7) and the mean number of logistical difficulties was 1.6 (SD 1.6, min 0, max 7).

In univariable analysis, study protocols sponsored by a public institution (hospital or University) and single center studies encountered more difficulties overall and more scientific difficulties in particular (Table 2). Compared to interventional designs, observational designs encountered more scientific difficulties, but fewer logistical difficulties. The presence of a statistician in the study team was associated with fewer scientific difficulties. Study protocols informing clinical questions (i.e., interventional, diagnostic or prognostic studies) were associated with more logistical difficulties compared to other types of research (studies of health/illness, public health, study methods, etc.). The same applied to the first REC decision: study protocols whose first REC decision either required an additional assessment or was negative (including non-consideration) reported more logistical difficulties compared to study protocols that received a positive first decision.

**Table 2:** Univariable associations (one-way ANOVA) with mean number of difficulties in clinical research

	All difficulties (15 items)		Scientific^a^ difficulties (7 items)		Logistical^b^ difficulties (7 items)	
	Mean	*p*-value	Mean	*p*-value	Mean	*p*-value
Study design		.45		.002		.047
Interventional	2.7		0.9		1.8	
Observational	2.9		1.4		1.4	
First decision of the REC		.25		.89		.022
Positive, positive with recommendations, positive with written modifications required	2.7		1.1		1.5	
Positive with conditions and reassessment, negative/non-consideration	3.1		1.1		2.0	
Origin of the study protocol		<.001		<.001		.12
Public (hospital, University, etc.)	3.1		1.4		1.6	
Private (industry, foundation)	1.6		0.2		1.3	
Sample size		.33		.092		.96
≤ median (100)	2.9		1.3		1.6	
> median	2.6		1.0		1.6	
Number of centers		<.001		<.001		.21
Single	3.2		1.5		1.7	
Multicenter	2.1		0.6		1.5	
Type of research		.79		.084		.006
Clinical (intervention, diagnosis, prognosis)	2.8		1.0		1.8	
Others (health/illness, public health/health economics, methods, other)	2.7		1.3		1.3	
Self-reported trainings in quantitative methods		.37		.16		.80
None	2.9		1.3		1.6	
Any	2.7		1.0		1.6	
Presence of a statistician on study team (*N* = 384)		.086		<.001		.54
No	2.9		1.3		1.6	
Yes	2.4		0.6		1.7	
Study published		.71		.90		.34
No	2.7		1.1		1.5	
Yes	2.8		1.1		1.6	

^a^Sum of difficulties reported on the following items: literature review, research question, design, data management, data analysis, manuscript writing, and publication in a peer-reviewed journal

^b^Sum of difficulties reported on the following items: approval by the Research Ethic Committee, getting funding, approval by the safety agencies, patient enrollment, data collection, collaboration with caregivers for patient enrollment, recruiting competent staff to conduct the study

In multivariable analysis, study design, first decision of the REC and the presence of a statistician on the study team were not associated with a higher number of difficulties (Additional file 1: Appendix 5) while several risk factors remained significantly associated. In a parsimonious model (Table 3), publicly sponsored and single center studies were associated with more difficulties overall and more scientific difficulties. Studies informing clinical questions were associated with higher number of difficulties overall and with more logistical difficulties.

**Table 3:** Multivariable linear regression of sum of difficulties in clinical research

	All difficulties (15 items)		Scientific difficulties (7 items)		Logistical difficulties (7 items)	
Study characteristics:	Difference	95 % CI	Difference	95 % CI	Difference	95 % CI
Public sponsorship^a^ (vs. private)	1.3	0.4,2.1	0.9	0.4,1.3	0.4	-0.1,0.9
Single center study (vs. multicenter)	0.8	0.1,1.5	0.6	0.2,1.0	0.3	-0.1,0.7
“Clinical” study^b^ (vs. other^c^)	0.7	0.0,1.3	0.1	-0.2,0.5	0.6	0.3,1.0

Difference = beta coefficients; CI = confidence interval

^a^Public hospital, University

^b^ Intervention or diagnosis or prognosis studies

^c^health/illness, public health/health economics, methods, other

### Specific difficulties by risk factors

The three study characteristics from the above multivariate model (private vs. public, multi vs. single center and “clinical” vs. other) were cross-tabulated with specific difficulties (Table 4). Compared to publicly sponsored studies, studies initiated by a private sponsor experienced fewer difficulties with the research question, study design, obtaining funding, data management, data analysis and interpretation, manuscript writing, and publication in a peer-reviewed journal. Similar results were observed when multi center studies were compared to single-center studies. Publicly sponsored studies and single center studies experienced fewer difficulties concerning the approval by safety agencies, however more difficulty with getting approval from the REC.

**Table 4:** Difficulties in clinical research by study characteristics^a^

	Study characteristics								
	Private^b^sponsorship	Public sponsorship		Multi center	Single center		Other^c^	Clinical^d^	
Difficulties with:	%	%	*p*	%	%	*p*	%	%	*p*
Literature review (get relevant articles, read, synthesize)	2.4	6.9	0.19	3.1	7.9	0.078	7.2	4.9	0.39
Research question (formulation of a main research question and/or hypothesis)	1.2	13.1	0.001	3.2	15.0	<0.001	15.1	5.9	0.004
Study design (identification of the study design, variables, instruments, analysis, including calculation of the sample size)	2.4	25.8	<0.001	8.2	29.1	<0.001	23.3	18.0	0.21
Approval by the Research Ethics Committee	12.2	17.9	0.25	11.9	20.3	0.038	12.8	20.4	0.056
Getting funding	3.7	23.9	<0.001	13.8	23.1	0.026	19.0	19.5	1.00
Approval by the safety agencies (Swiss Agency for Therapeutic Products, etc.)	17.3	8.3	0.023	14.6	7.0	0.024	3.9	15.7	<0.001
Patients enrollment	51.3	41.5	0.13	48.1	42.5	0.30	37.1	51.5	0.005
Data collection	19.5	28.9	0.12	22.0	30.0	0.10	26.1	27.2	0.82
Collaboration with caregivers for patient enrollment	18.3	22.1	0.54	20.1	21.8	0.80	19.4	22.5	0.53
Recruitment of competent persons to conduct the study	12.2	22.0	0.059	16.4	22.9	0.12	16.7	23.3	0.13
Data management	6.1	20.4	0.002	10.1	21.8	0.002	19.6	14.6	0.22
Data analysis (statistical or other) and results interpretation	3.7	26.7	<0.001	10.8	29.3	<0.001	23.5	20.1	0.46
Writing a manuscript for a scientific publication	1.2	23.8	<0.001	8.9	25.6	<0.001	20.9	16.7	0.36
Publication in a peer-reviewed journal	4.9	25.5	<0.001	14.6	24.8	0.020	21.1	20.0	0.80
Other(s) difficulty(ies)	3.7	5.8	0.58	5.0	5.3	1.00	8.9	1.9	0.002

^a^Selected from the multivariable model (Table 3). *P*-values come from the Fisher’s exact test

^b^Industry or foundation

^c^Health/illness, public health, methodology, etc

^d^Intervention or diagnosis or prognosis

Furthermore, studies informing clinical questions reported more difficulties with approval by safety agencies or with patient enrollment. Non-clinical studies reported more difficulties with the formulation of the research question.

## Discussion

The present study describes difficulties experienced in the course of a specific study by a broad sample of medical researchers at a Swiss university hospital. The main finding is that patient enrollment posed a significant difficulty to 44 % of study protocols, and data collection posed difficulty to 27 % of them. In other words, logistical aspects of the research process seem to be the major sources of problems in medical research. In contrast, formulating the research question and performing the literature review were less likely to be an issue for medical researchers. Across the whole research process, medical researchers experienced on average three (2.8) of 14 difficulties and only one out of four reported no difficulty at all in conducting their study.

In a previous study investigating perceived barriers in general, a majority of medical researchers reported numerous obstacles in conducting research [19]. We found lower prevalence rates, probably because we assessed difficulties in a given study, and not over the career of a researcher. Furthermore, we only included completed studies, which did therefore not encounter a “fatal” problem.

The correlations between 14 types of difficulties suggested two distinct dimensions: first, problems with the logistics of a study, from funding to patient enrollment and research personnel issues; second, scientific problems. Thus, different kinds of interventions are probably needed to facilitate the conduct of clinical research. Logistical problems could be alleviated by a core facility that would provide administrative support, fund-raising assistance, and qualified research associates. Scientific problems could be solved by the provision of statistical assistance and methodological training of clinical researchers. Such interventions have been implemented with success in other settings [36]. It should be noted that such structures were set up only in 2007 at the hospital under study, most likely too late to have any major influence on the course of research protocols approved between 2001 and 2005. The provision of statistical assistance may also have various impacts on the number of patients to enroll [37]: one the one hand, statistical assistance could increase the sample size at the stage of designing the study; on the other hand, statistical assistance could help medical researchers determine the smallest necessary sample size. Thus, it is uncertain whether the provision of statistical assistance would reduce or increase logistical problems. In our results (multivariable model), a statistician in the team was not associated with logistical difficulties.

Studies initiated by a public sponsor (hospital or university) as well as single center studies were associated with more difficulties compared to those with private sponsor or those multi center: these associations were significant for the overall and the scientific scores, but not for the logistical score. When the study protocol came from a public sponsor and when it was a single center study, it is more likely that the PI was in charge of most steps, from conception to publication. In contrast, many of these tasks are typically not conducted by hospital based PIs when study protocols are initiated by the industry.

Investigators of studies addressing clinical questions also encountered more logistical difficulties, probably because they need to enroll and collect data from patients. Most often, patient recruitment is conducted in parallel to the delivery of healthcare, thus requiring complex and numerous interactions with healthcare providers. Although as many as 72 % of patients invited to take part in a clinical study in our institution accept to do so [38], a better coordination of projects enrolling similar patients in parallel might facilitate recruitment. Other challenges relate to the patients’ willingness to participate, which will depend on personal factors (such as level of education), but also on the attributes of the study. As shown in a previous work, patients are more willing to participate in studies that explicitly value safety, convenience, appropriate oversight, and open communication [39].

In contrast with other surveys [18, 19], we found that getting funding did not represent a significant barrier in conducting clinical research. Three explanations can be considered: first, we surveyed medical researchers about completed studies (which were all funded), while other surveys assessed the general opinion of medical researchers. Second, Switzerland is a relatively favorable country for conducting research: between 2000 and 2008, the funding for research and development increased from 2.5 % of the gross domestic product to 3.0 % [40]. Third, in some cases, clinical studies can be conducted without external funding when benefiting from non-financial support of the hospital.

Another result worth noting is the similarity in the average number of difficulties encountered by PI trained in quantitative methods compared to those not trained in these methods. Training in quantitative methods probably has limited impact on logistical issues; however we expected an impact on scientific issues. It is possible that trained investigators design and conduct more complex or ambitious studies, thus facing a similar absolute number of challenges, yet at a more advanced level.

More than one fourth (26.4 %) of study protocols were conducted without difficulties. More research on these study protocols, using qualitative methods, could add evidence on the facilitating factors for conducting clinical research.

### Limitations

This study has several limitations. First, it was carried out at a single Swiss university hospital and we do not know to what extent the findings apply to other settings. Second, we only surveyed PIs of completed clinical studies, which may have decreased the apparent prevalence of major difficulties, because abandoned clinical studies [25, 29] may have experienced more difficulties. Third, PIs’ answers may have been affected by various recall biases [38]. Social desirability may also have influenced responses, considering that the investigators in charge of the present study belong to the same institution as the surveyed PIs. Fourth, the scale of difficulties (and the two subscales, logistic and scientific difficulties) was built for the purpose of this study, based on literature review and expert consensus. We have no evidence of their validity beyond face validity, though their reliability was acceptable (Cronbach alpha between 0.73 and 0.83). Moreover, among the 14 items composing the scale, four items (getting funding, data collection, recruitment of competent staff to conduct the study, and data management) shared variance on both subscales (see Additional file 1: Appendix 1). Fifth, the analysis of studies with no questionnaire suggested some evidence for non-response bias: undocumented studies were more frequently interventional studies, with industry sponsorship, and unpublished.

## Conclusions

Patient enrollment and logistical problems topped the list of difficulties. Clinical studies and single-center studies were at higher risk of experiencing logistical difficulties. Providing both logistical and methodological support to researchers may facilitate the conduct of research projects.

## Ethical approval

This study was approved by the REC of the Geneva University Hospitals.

## Availability of data and materials

The data are not available online but can be requested to the corresponding author.

## Additional file

Additional file 1: Appendix 1. Components of factor analysis, with varimax rotation. Appendix 2 Characteristics^1^ of the 408 studies with no follow-up questionnaire returned, and differences with included studies. Appendix 3 Number of self-reported difficulties* in clinical research by study protocol. Appendix 4 Spearman correlation matrix for sum of difficulties in clinical research and study characteristics. Appendix 5 Full model of sum of difficulties in clinical research by study characteristics (Multivariable linear regression). (DOCX 26 kb)

## Abbreviations

REC: Research Ethics Committee
PIs: principal investigators
SD: standard deviation
