# Supplementary material for: Patient enrollment and logistical problems top the list of difficulties in clinical research: a cross-sectional survey
Source: BMC Med Res Methodol. 2016 May 4;16:50. doi: 10.1186/s12874-016-0151-1 (PMC4855713; doi:10.1186/s12874-016-0151-1)
Supplement: Additional file 1: — Appendix 1. Components of factor analysis, with varimax rotation. Appendix 2 Characteristics1 of the 408 studies with no follow-up questionnaire returned, and differences with included studies. Appendix 3 Number of self-reported difficulties* in clinical research by study protocol. Appendix 4 Spearman correlation matrix for sum of difficulties in clinical research and study characteristics. Appendix 5 Full model of sum of difficulties in clinical research by study characteristics (Multivariable linear regression). (DOCX 26 kb) [file 12874_2016_151_MOESM1_ESM.docx]

Additional file

Appendix 1 Components of factor analysis, with varimax rotation

|  | Factors^a^ | |
| --- | --- | --- |
|  | 1 | 2 |
| Literature review | .574 |  |
| Research question | .773 |  |
| Study design | .774 |  |
| Approval by the REC |  | .626 |
| Getting funding | .308 | .472 |
| Approval by the safety agencies |  | .545 |
| Patients enrollment |  | .654 |
| Data collection | .465 | .608 |
| Collaboration with caregivers for patient enrollment |  | .684 |
| Recruitment of competent staff to conduct the study | .350 | .503 |
| Data management | .601 | .456 |
| Data analysis and results interpretation | .759 |  |
| Writing a manuscript for a scientific publication | .799 |  |
| Publication in a peer-reviewed journal | .698 |  |

^a^ value less 0.3 are not reported

Appendix 2 Characteristics^1^ of the 408 studies with no follow-up questionnaire returned, and differences with included studies.

|  | N(%) | Difference (p-value)^2^ with included studies |
| --- | --- | --- |
| Study design |  | 0.026 |
| Interventional | 220 (53.9) |  |
| Observational | 188 (46.1) |  |
| First decision of the research ethics committee (N=402) |  | 0.27 |
| Positive, positive with recommendations, or positive with written modifications required | 331 (82.3) |  |
| Positive with conditions and reassessment, or negative and non-considered, but approved at a later stage | 71 (17.7) |  |
| Financial sponsorship of the study protocol (N=400) |  | 0.003 |
| Public (hospital, university) | 269 (67.3) |  |
| Private (industry, foundation) | 121 (30.3) |  |
| Both | 10 (2.5) |  |
| Link with the industry (N=395) |  | 0.004 |
| None | 236 (59.7) |  |
| Industry with indication of support (funding, drugs, human resources) | 141 (35.7) |  |
| Industry without indication of support | 18 (4.6) |  |
| Sample size (N=403) |  | 0.98 |
| ≤100 | 227 (56.3) |  |
| >100 | 176 (43.7) |  |
| Sample size calculation (N=398) |  | 0.47 |
| Yes | 165 (41.5) |  |
| No | 233 (58.5) |  |
| Number of centers (N=403) |  | 0.26 |
| Single | 223 (55.3) |  |
| Multi center | 180 (44.7) |  |
| Research topic (N=405) |  | 0.12 |
| Health or illness (organs, tissues, cells, receptors, etc.) | 108 (26.7) |  |
| Intervention or treatment | 193 (47.7) |  |
| Diagnostics method | 29 (7.2) |  |
| Prognosis | 11 (2.7) |  |
| Public health, health economics, medical ethic, attitudes | 25 (6.2) |  |
| Research methods (questionnaires, indicators, measures, etc.) | 29 (7.2) |  |
| Other, unspecified topic (registries, medical record databases) | 10 (2.5) |  |
| Clinical specialty (N=404) |  | 0.001 |
| General | 31 (7.7) |  |
| Psychiatry | 28 (6.9) |  |
| Cardiology | 30 (7.4) |  |
| Oncology | 64 (15.8) |  |
| Infectious diseases | 32 (7.9) |  |
| Neurology | 24 (5.9) |  |
| Rheumatology | 5 (1.2) |  |
| Other clinical specialties | 159 (39.4) |  |
| Two or more specialties | 31 (7.7) |  |
| Presence of a statistician in the study team (N=401) |  | 0.45 |
| Yes | 112 (27.9) |  |
| No | 289 (72.1) |  |
| Study published |  | <0.001 |
| Yes | 183 (44.9) |  |
| No | 225 (55.1) |  |

^1^ Information from study protocols stored in the REC’ archives

^2^ Chi-square p-value

Appendix 3 Number of self-reported difficulties* in clinical research by study protocol

|  | N (%) |
| --- | --- |
| No difficulty | 102 (26.4) |
| 1 difficulty | 72 (18.7) |
| 2 difficulties | 49 (12.7) |
| 3 difficulties | 42 (10.9) |
| 4 difficulties | 26 (6.7) |
| 5 difficulties | 25 (6.5) |
| 6 difficulties | 18 (4.7) |
| 7 difficulties | 14 (3.6) |
| 8 difficulties | 19 (4.9) |
| 9 difficulties | 9(2.3) |
| 10 difficulties | 6 (1.6) |
| 11 difficulties | 3 (0.8) |
| 12 difficulties | 1 (0.3) |
| *rated medium or great or very great among 14 difficulties related to the research process (see list in Table4) | |

Appendix 4 Spearman correlation matrix for sum of difficulties in clinical research and study characteristics

|  | All diff | SD | LD | Design | REC | Origin | Sample | Centers | Type | Skills | Stat |
| --- | --- | --- | --- | --- | --- | --- | --- | --- | --- | --- | --- |
| All diff | - |  |  |  |  |  |  |  |  |  |  |
| SD | 0.811** | - |  |  |  |  |  |  |  |  |  |
| LD | 0.874** | 0.477** | - |  |  |  |  |  |  |  |  |
| Design | 0.008 | -0.147* | 0.110* | - |  |  |  |  |  |  |  |
| REC | 0.089 | 0.017 | 0.128* | 0.059 | - |  |  |  |  |  |  |
| Origin | 0.205** | 0.322** | 0.082 | -0.458** | -0.083 | - |  |  |  |  |  |
| Sample | -0.046 | -0.108* | -0.002 | 0.109* | 0.097 | -0.243** | - |  |  |  |  |
| Centers | 0.156* | 0.284** | 0.055 | -0.311** | -0.067 | 0.526** | -0.296** | - |  |  |  |
| Type | 0.046 | -0.084 | 0.158* | 0.615** | 0.134* | -0.339** | 0.086 | -0.303** | - |  |  |
| Skills | -0.081 | -0.035 | -0.113 | -0.042 | -0.172* | -0.065 | -0.136* | -0.105 | -0.080 | - |  |
| Stat | -0.057 | -0.214** | 0.039 | 0.274** | 0.001 | -0.493** | 0.269** | -0.355** | 0.185** | 0.065 | - |
| Pub | -0.026 | -0.011 | 0.013 | 0.013 | 0.018 | 0.048 | 0.087 | -0.018 | 0.024 | 0.007 | -0.018 |

* p≤0.05, **p≤0.001

Acronyms: “All diff” Sum of all difficulties (15 items), “SD” Scientific difficulties (7 items), “LD” Logistical difficulties (7 items), “Design” Study design (interventional vs observational), “REC” First decision of the REC, “Origin” Origin of the study protocol (public vs. private sponsorhip), “Sample” Sample size (≤ median vs. > median), “Centers” Number of centers (single vs. multi centers), “Type” Type of research (clinical study vs. other), “Skills” Self-reported trainings in quantitative methods, “Stat” Presence of a statistician on study team, “Pub” Study published.

Appendix 5 Full model of sum of difficulties in clinical research by study characteristics (Multivariable linear regression)

|  | All difficulties  (15 items) | | Scientific difficulties  (7 items) | | Logistical difficulties  (7 items) | |
| --- | --- | --- | --- | --- | --- | --- |
| Study characteristics: | Difference | 95% CI | Difference | 95% CI | Difference | 95% CI |
| Interventional (vs. observational) | 0.2 | -0.6, 1.0 | -0.1 | -0.6, 0.3 | 0.2 | -0.2, 0.7 |
| First REC decision reassessment or negative (vs. positive) | 0.4 | -0.3, 1.2 | 0.1 | -0.4, 0.5 | 0.4 | -0.0, 0.8 |
| Public sponsorship^1^ (vs. private) | 1.5 | 0.5, 2.4 | 0.8 | 0.2, 1.3 | 0.7 | 0.2, 1.2 |
| Single center study (vs. multi center) | 0.8 | 0.1, 1.5 | 0.6 | 0.2, 1.0 | 0.2 | -0.2,0.6 |
| “Clinical” study^2^ (vs. other^3^) | 0.5 | -0.2, 1.3 | 0.2 | -0.2,0.6 | 0.4 | 0.0, 0.8 |
| Statistician on study team (vs. no) | 0.3 | -0.5, 1.0 | -0.1 | -0.6, 0.3 | 0.4 | -0.1,0.8 |
| Difference= beta coefficients; CI=confidence interval  ^1^ Public hospital, University; ^2^ Intervention or diagnosis or prognosis studies; ^3^ health/illness, public health/ health economics, methods, other | | | | | | |
